# Lung function over the life course of paediatric and adult patients with cystic fibrosis from a large multi-centre registry

**DOI:** 10.1038/s41598-020-74502-1

**Published:** 2020-10-15

**Authors:** Arul Earnest, Farhad Salimi, Claire E. Wainwright, Scott C. Bell, Rasa Ruseckaite, Tom Ranger, Tom Kotsimbos, Susannah Ahern

**Affiliations:** 1 School of Public Health and Preventive Medicine, Monash University, Room 415, Level 4, 553 St. Kilda Road, Melbourne, VIC 3004 Australia; 2 Faculty of Medicine, University of Queensland, Brisbane, Australia; 3 Department of Respiratory and Sleep Medicine, Queensland Children’s Hospital, Brisbane, Australia; 4 Translational Research Institute, Brisbane, Australia; 5 Department of Thoracic Medicine, The Prince Charles Hospital, Brisbane, Australia; 6 Department Respiratory Medicine, Alfred Hospital, Melbourne, Australia

**Keywords:** Medical research, Risk factors

## Abstract

A key measure of lung function in people with Cystic Fibrosis (CF) is Forced Expiratory Volume in the first second FEV_1_ percent predicted (FEV_1_pp). This study aimed to address challenges in identifying predictors of FEV_1_pp, specifically dealing with non-linearity and the censoring effect of death. Data was obtained from a large multi-centre Australian Cystic Fibrosis Data Registry (ACFDR). A linear mixed model was used to study FEV_1_pp as the endpoint. There were 3655 patients (52.4% male) included in our study. Restricted cubic splines were used to fit the non-linear relationship between age of visit and FEV_1_pp. The following predictors were found to be significant in the multivariate model: age of patient at visit, BMI z-score, age interaction with lung transplantation, insulin dependent diabetes, cirrhosis/portal hypertension, pancreatic insufficiency, *Pseudomonas aeruginosa* infection and baseline variability in FEV_1_pp. Those with *P. aeruginosa* infection had a lower mean difference in FEV_1_pp of 4.7 units, *p* < 0.001 compared to those who did not have the infection. Joint modelling with mortality outcome did not materially affect our findings. These models will prove useful for to study the impact of CFTR modulator therapies on rate of change of lung function among patients with CF.

## Introduction

Cystic fibrosis (CF) is an autosomal disease caused by mutations in the cystic fibrosis transmembrane conductance regulator (*CFTR*) gene. In 2017 the Australian CF data registry (ACFDR) recorded 3151 patients to have CF with 54% being adult, a median age of 19.6 years, and 54% male^1^. Significant clinical advances have prolonged the average life expectancy for patients with CF and the development of CFTR modulator therapies will also impact disease progression. Despite improved outcomes, lung disease progression will still lead to respiratory failure, and monitoring of lung function and appropriate referral for lung transplantation will remain extremely important^2^.

The main measure of lung function in people with CF is forced expiratory volume in one second percentage predicted (FEV_1_pp). One of the main goals of clinical care is to minimise the loss of lung function (decrease in FEV_1_pp) across the patients’ lifespan. Understanding the natural trajectory of FEV_1_pp in people with CF is essential to direct early intervention and prevent progression of lung damage, and to inform referral of patients for lung transplantation and novel therapeutic strategies^3^. Longitudinal statistical models, accounting for known predictors such as patient sex, height and genotype, and based on clinical data, provide a means to predict the course of lung function in patients and therefore inform clinical decision making^4^. There have been several attempts to study FEV_1_pp changes in people with CF using different methodologies and a recent review of articles has identified gaps and concluded that “further longitudinal studies are needed to develop statistical models to deal with nonlinear changes in lung function, quantify variability and the influence of risk factors. This should be undertaken without ignoring the censoring effect of death or lung transplantation”^4^.

Since 1998, the ACFDR has collected diagnostic and treatment data from more than 90 percent of the population of people with CF in Australia^1^. Data about a CF patient’s diagnosis, treatment and complications are collected by the registry through the treating sites so as to improve health service provision and better comprehend the treatment of CF and outcomes for patients. The ACFDR is managed by Monash University as part of a portfolio of approximately 30 clinical quality registries and is funded by Cystic Fibrosis Australia with the CF specialist centres contributing data. The main objective of our study was to provide a holistic FEV_1_pp modelling approach dealing with different slopes for before and after lung transplantation, handling death as an outcome and optimally dealing with the issue of non-linearity between FEV_1_pp and age of visit.

## Methods

### Data

The main source of data for our analysis was de-identified patient-level records by visit dates from the ACFDR from 2008–2019. People are registered at the time they are diagnosed with CF unless they do not consent. For minors, parental/ guardian consent is required. The ACFDR contains detailed demographic and clinical information of patients with a confirmed diagnosis of CF, and who receive clinical care at 23 specialist CF centres in Australia^1^. We excluded visits where patients were aged less than 6 years, as FEV_1_ may not be as reliable or consistently measured for these younger patients and hence the data may not be epidemiologically robust. We also excluded patients who had only one FEV_1_pp measurement across the study period. FEV_1_pp was calculated from the raw FEV_1_ scores using the Global Lung Initiative(GLI) established prediction equations.

To improve the quality of the registry, transplantation data linkage was conducted with the Australia and New Zealand Cardiothoracic Transplant Registry (ANZCOTR). Additional information on the type and date of the transplants was sought from the individual centres. Accurate survival information is an important outcome of the Registry, providing an understanding of the impact of quality improvements in practice and care over time. To ensure accuracy of the survival data, a linkage with the National Death Index was undertaken^1^. Patient names are not held by the registry, so linkage was based on probabilistic matching, using patient initials, date of birth, gender and patient’s residential postcode.

Ethics approval for this study was provided by Monash University Human Research Ethics Committee (approval number: 16397). Informed consent was obtained from individuals and families of those under 18 years of age, for sites where ethics committee required and an opt-out consent was applied to the other sites where patients had the opportunity to contact the registry and opt out from the registry. All methods were carried out in accordance with relevant guidelines and regulations.

### Statistical methods

Our primary endpoint was FEV_1_pp, which was calculated for each patient visit where FEV_1_ was measured. Several different statistical approaches have been used to study the relationship between important risk factors and CF lung disease progression as measured by FEV_1_pp. Current statistical models include generalised linear models (GLM) (which are unable to account for within-patient correlation in the data)^5–10^, generalised estimating equations (GEE) (which do account for within-patient clustering, but are population averaged and therefore unable to model individual patient’s trajectories in FEV_1_pp)^11–17^ and linear mixed models, which address the limitations mentioned above as well as allowing both fixed effect covariates and random intercepts to be modelled (e.g. treating centre effects)^18–32^.

A linear mixed model^4^ was used to determine changes in FEV_1_pp over age of visit. A two-way random intercept term was fitted to capture both treating centre effects as well as within-patient variation in FEV_1_pp. A random slope term was specified at the patient level, so that each patient was allowed to have their own trajectory of FEV_1_pp over time. Unstructured covariance was specified to allow for distinct correlations between the random intercept and slope terms.

Univariate models were fit for each of these covariates: age of patient at visit, gender, Body Mass Index (BMI) z-score grouped in quartiles, age at diagnosis, patient genotype, lung transplant status, pancreatic insufficiency, birth cohort, insulin dependent diabetes, cirrhosis or portal hypertension, *Pseudomonas aeruginosa* infection and baseline variability in FEV_1_pp. As there was a non-linear relationship between FEV_1_pp and age of patient at visit, we fitted restricted cubic splines, carefully evaluating various knots and knot locations and used the Akaike information criterion (AIC) to select the optimal combination. Pancreatic insufficiency was defined as presence of clinical symptoms (fecal fat test, steatorrhoea) and administration of pancreatic replacement enzymes. *P. aeruginosa* infection was defined as at least one positive test during followup. Baseline variability in FEV_1_pp was calculated as the difference between the highest FEV_1_pp and the median FEV_1_pp during the subjects’ first 2 years of followup^33^.

Starting from the most significant covariate identified in the univariate analysis, we used the likelihood ratio test to see whether the inclusion of the next most significant variable helped to significantly improve the fit of the model. This was done sequentially until we evaluated all the variables. For the final multivariate model, to account for different slopes for lung transplantation, we specified separate slopes for FEV_1_pp before/ after transplantation by including appropriate interaction terms in the model. We plotted separate adjusted means for the variables in the final multivariate model using the margins command, where all the other covariates were kept at their mean values and we specified interaction terms between the cubic splines and the covariates.

To deal with the impact of mortality on our final multivariate results, we undertook a joint longitudinal and survival model using the *‘jmxtstcox’* in Stata which fits a cox proportional hazards model and a linear mixed model with a random intercept, assuming that they share common random intercepts^34^. As this is a new command still under development by Stata Corp, we opted to include the results as a supplementary analysis in our manuscript. Survival time was defined as time from birth till death or censored at 31 December 2019 for those still alive. We also undertook sensitivity analyses for the multivariate model in order to test the robustness our models, particularly relating to the impact of potential outliers and excluding lung transplantation variable from the final model. Data analysis was performed in Stata V16 (Collage Station, Tx, USA) and level of significance set at 5%.

## Results

### Descriptives

Out of the original 188,394 visits in the study period, after excluding patient visits before 6 years of age, and those with missing or only 1 FEV_1_pp measurement across the whole study period, the final number of patients included in our analysis was 3655 (100,907 visits). The median duration of follow-up between birth and last visit was 21.7 years (Interquartile range IQR: 14.9–32.2 years) for the entire cohort.

Table 1 highlights the demographic and clinical characteristics of all patients. Of the 3655 patients, slightly more than half (52.4%) were male. The mean age at last visit was 24.6 ± 12.8 years. Around 27.4% were underweight at the last visit and around 55.3% presented with normal weight. The median age of diagnosis was 0.1 years (IQR: 0.05–0.4 years). 18.1% had insulin dependent diabetes and 6.5% had cirrhosis or portal hypertension. The prevalence of *P. aeruginosa* infection was 50.3% while 9.6% of patients had a lung transplant and 7.8% of the entire cohort died. There were significant differences in the demographic and clinical variables across age group at visit (Table 1).

**Table 1:** Demographic and clinical characteristics of cystic fibrosis patients.

Age group at visit	6–12	13–17	18 +	All	*p* value
N	708	595	2352	3655	
Sex					0.33
Male	354 (50.0%)	311 (52.3%)	1250 (53.2%)	1915 (52.4%)	
Female	354 (50.0%)	284 (47.7%)	1101 (46.8%)	1739 (47.6%)	
Age at visit in years, mean (SD)	9.79 (1.97)	15.71 (1.52)	31.32 (11.02)	24.61 (12.79)	< 0.001
BMI group at visit in kg/m^2^					< 0.001
Underweight	545 (77.0%)	191 (32.1%)	266 (11.3%)	1002 (27.4%)	
Normal	152 (21.5%)	357 (60.0%)	1512 (64.3%)	2021 (55.3%)	
Overweight	11 (1.6%)	35 (5.9%)	441 (18.8%)	487 (13.3%)	
Obese	0 (0.0%)	12 (2.0%)	110 (4.7%)	122 (3.3%)	
Unknown	0 (0.0%)	0 (0.0%)	23 (1.0%)	23 (0.6%)	
BMI z-score	0.06 (0.89)	− 0.30 (1.11)	− 1.34 (13.60)	− 0.37 (6.31)	0.003
Age at diagnosis in years, median (IQR)	0.08 (0.04, 0.14)	0.10 (0.05, 0.15)	0.16 (0.07, 1.46)	0.12 (0.05, 0.42)	< 0.001
FEV_1_/FVC ratio, mean (SD)	0.83 (0.08)	0.78 (0.12)	0.66 (0.15)	0.71 (0.15)	< 0.001
FEV_1_%predicted GLI , mean (SD)	90.41 (17.26)	79.64 (22.95)	62.38 (26.05)	70.62 (26.70)	< 0.001
Lung transplant					< 0.001
No	704 (99.4%)	563 (94.6%)	2036 (86.6%)	3303 (90.4%)	
Yes	4 (0.6%)	32 (5.4%)	316 (13.4%)	352 (9.6%)	
Died					< 0.001
No	704 (99.4%)	551 (92.6%)	2116 (90.0%)	3371 (92.2%)	
Yes	4 (0.6%)	44 (7.4%)	236 (10.0%)	284 (7.8%)	
Age at death, mean (SD)	10.71 (1.99)	18.24 (2.63)	34.55 (12.78)	31.69 (13.34)	< 0.001
Pancreatic insufficiency					< 0.001
Yes	550 (77.7%)	478 (80.3%)	1701 (72.4%)	2729 (74.7%)	
No	146 (20.6%)	102 (17.1%)	461 (19.6%)	709 (19.4%)	
Unknown	12 (1.7%)	15 (2.5%)	187 (8.0%)	214 (5.9%)	
Patient genotype					< 0.001
F508del/F508del	336 (47.7%)	311 (52.4%)	1069 (45.5%)	1716 (47.1%)	
F508del/other	262 (37.2%)	188 (31.6%)	695 (29.6%)	1145 (31.4%)	
Other/other	40 (5.7%)	23 (3.9%)	179 (7.6%)	242 (6.6%)	
Unknown	67 (9.5%)	72 (12.1%)	405 (17.2%)	544 (14.9%)	
Birth cohort year					< 0.001
Before 1998	2 (0.3%)	109 (18.3%)	2023 (86.0%)	2134 (58.4%)	
1998/2013	706 (99.7%)	486 (81.7%)	329 (14.0%)	1521 (41.6%)	
Insulin dependent diabetes					< 0.001
No/unknown	677 (95.6%)	493 (82.9%)	1821 (77.5%)	2991 (81.9%)	
Yes	31 (4.4%)	102 (17.1%)	530 (22.5%)	663 (18.1%)	
Cirrhosis or portal hypertension					< 0.001
No/unknown	694 (98.0%)	559 (93.9%)	2165 (92.1%)	3418 (93.5%)	
Yes	14 (2.0%)	36 (6.1%)	186 (7.9%)	236 (6.5%)	
Mean baseline variability in FEV_1_pp	9.38 (7.22)	8.77 (6.48)	7.25 (6.68)	7.91 (6.82)	< 0.001
*Pseudomonas aeruginosa* infection					< 0.001
No/unknown	446 (63.0%)	374 (62.9%)	995 (42.3%)	1815 (49.7%)	
Yes	262 (37.0%)	221 (37.1%)	1356 (57.7%)	1839 (50.3%)	

FVC—Forced Vital Capacity; FEV_1_—Forced Expiratory Volume in the 1st second; FEV_1_% predicted GLI—Forced Expiratory Volume in the 1st second percent predicted (Global Lung function Initiative method). BMI—Body Mass Index; IQR- Interquartile range; Note: for all time-varying covariates such as age, BMI, FVC and FEV_1_, data is presented for the last visit. All *p* values from Chi-squared test, unless otherwise indicated. *p* values for age of visit, BMI z-score, age at death, FEV_1_pp, FEV_1_/FVC ratio and baseline variability in FEV_1_pp from Analysis of Variance test. *p* value for age at diagnosis from Kruskal–Wallis test.

**Figure 1:**
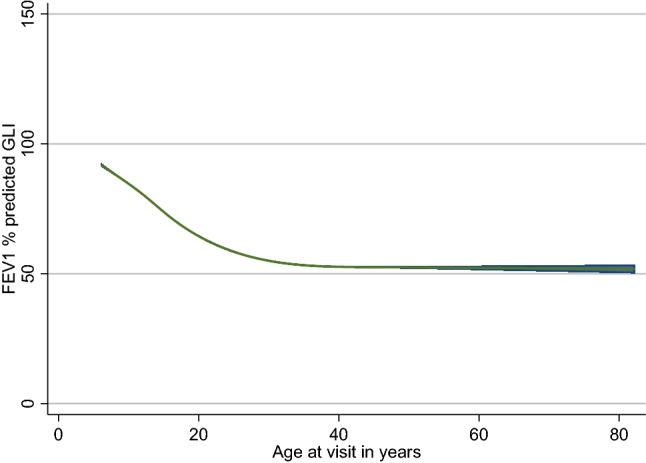
Restricted cubic splines showing non-linear relationship between FEV_1_ percent predicted and age at visit.

We found that there was a non-linear relationship between FEV_1_pp and age at visit (Fig. 1). There was an initial steeper non-linear decrease in FEV_1_pp from age 6 up till 30, and then a more gradual linear decline thereafter. Between ages 6 and 18, the rate of decline ranged from − 2.2 to − 1.8 units/ year. From 19 to 30 years, the rate varied from − 1.8 to − 0.5 units, and for those aged more than 30 years, the rate of decline was − 0.5 to 0.06 units.

### Univariate predictors of FEV_1_pp

We found that the model with random intercepts for patient and centre, along with a random slope for age at visit, provided a good fit to the data. We also found that a restricted cubic spline model for age of patient at visit with 3 knots fitted at ages 12, 18 and 30 provided a good fit to the model with the lowest AIC of 723,447 (Supplementary Information Table S1). In the univariate analysis, we found the following factors to be associated with FEV_1_pp: Age of visit, BMI z-score, pancreatic insufficiency, patient genotype, lung transplantation, birth cohort, insulin dependent diabetes, cirrhosis or portal hypertension, *P. aeruginosa* infection and baseline variability in FEV_1_pp (Table 2). Gender and age of diagnosis were non-significant.

**Table 2:** Univariate factors associated with FEV_1_ percent predicted.

Covariates	Coefficient	95% CI		*p* value
Restricted cubic spline, age of visit (6–12 years)	− 0.51	− 0.67	− 0.34	< 0.001
Restricted cubic spline, age of visit (13–18 years)	− 35.84	− 41.75	− 29.93	< 0.001
Restricted cubic spline, age of visit (19–30 years)	84.98	70.47	99.50	< 0.001
Restricted cubic spline, age of visit (> 30 years)	− 54.35	− 65.29	− 43.41	< 0.001
Gender				
Male (n = 1915)	Reference			
Female (n = 1739)	0.58	− 1.12	2.27	0.504
BMI z-score quartiles				
1st Quartile	Reference			
2nd Quartile	3.14	2.90	3.39	< 0.001
3rd Quartile	5.44	5.14	5.73	< 0.001
4th Quartile	7.06	6.69	7.42	< 0.001
Unknown	0.96	0.55	1.36	< 0.001
Age at diagnosis (years) (n = 2873)	− 0.09	− 0.29	0.10	0.359
Mortality				
Alive (n = 3371)	Reference			
Dead (n = 284)	− 17.05	− 20.33	− 13.77	< 0.001
Pancreatic insufficiency				
No (n = 709)	Reference			
Yes (n = 2729)	− 6.33	− 8.54	− 4.13	< 0.001
Unknown (n = 214)	− 4.94	− 9.51	− 0.37	0.034
Patient genotype				
F508del/F508del (n = 1716)	Reference			
F508del/Other (n = 1145)	4.61	2.69	6.54	< 0.001
G551d/ G551d (n = 242)	− 1.02	− 4.76	2.71	0.591
Unknown (n = 544)	− 2.45	− 5.09	0.19	0.069
Lung Transplant				
No (n = 3303)	Reference			
Yes (n = 352)	− 25.25	− 28.27	− 22.22	< 0.001
Birth cohort year				
Before 1998 (n = 2134)	Reference			
1998/2013 (n = 1521)	11.70	9.98	13.41	< 0.001
Insulin dependent diabetes				
No/unknown (n = 2991)	Reference			
Yes (n = 663)	− 10.22	− 12.35	− 8.09	< 0.001
Cirrhosis or portal hypertension				
No/unknown (n = 3418)	Reference			
Yes (n = 236)	− 7.35	− 10.61	− 4.10	< 0.001
*Pseudomonas aeruginosa* infection				
No/unknown (n = 1815)	Reference			
Yes (n = 1839)	− 8.64	− 10.38	− 6.89	< 0.001
Baseline variability in FEV1pp (n = 3654)	− 0.54	− 0.67	− 0.42	< 0.001

There was a non-linear decline in FEV_1_pp across age of visit. We also found a dose–response relationship between the BMI z-score quartiles and FEV_1_pp. Those who were in the second, third and fourth quartile had a higher 3.1, 5.4 and 7.1 unit difference in FEV_1_pp as compared to those in the first quartile. A larger variability in baseline FEV_1_pp was associated with lower FEV_1_pp scores (− 0.5, 95% CI: − 0.7 to − 0.4), *p* < 0.001. Patients born in the most recent era between 1998 and 2013 had a higher FEV_1_pp value of 11.7 units (95% CI: 10.0 to 13.4) when compared to those born in an earlier era before 1998 (*p* < 0.001).

### Multivariate predictors of FEV_1_pp

**Figure 2:**
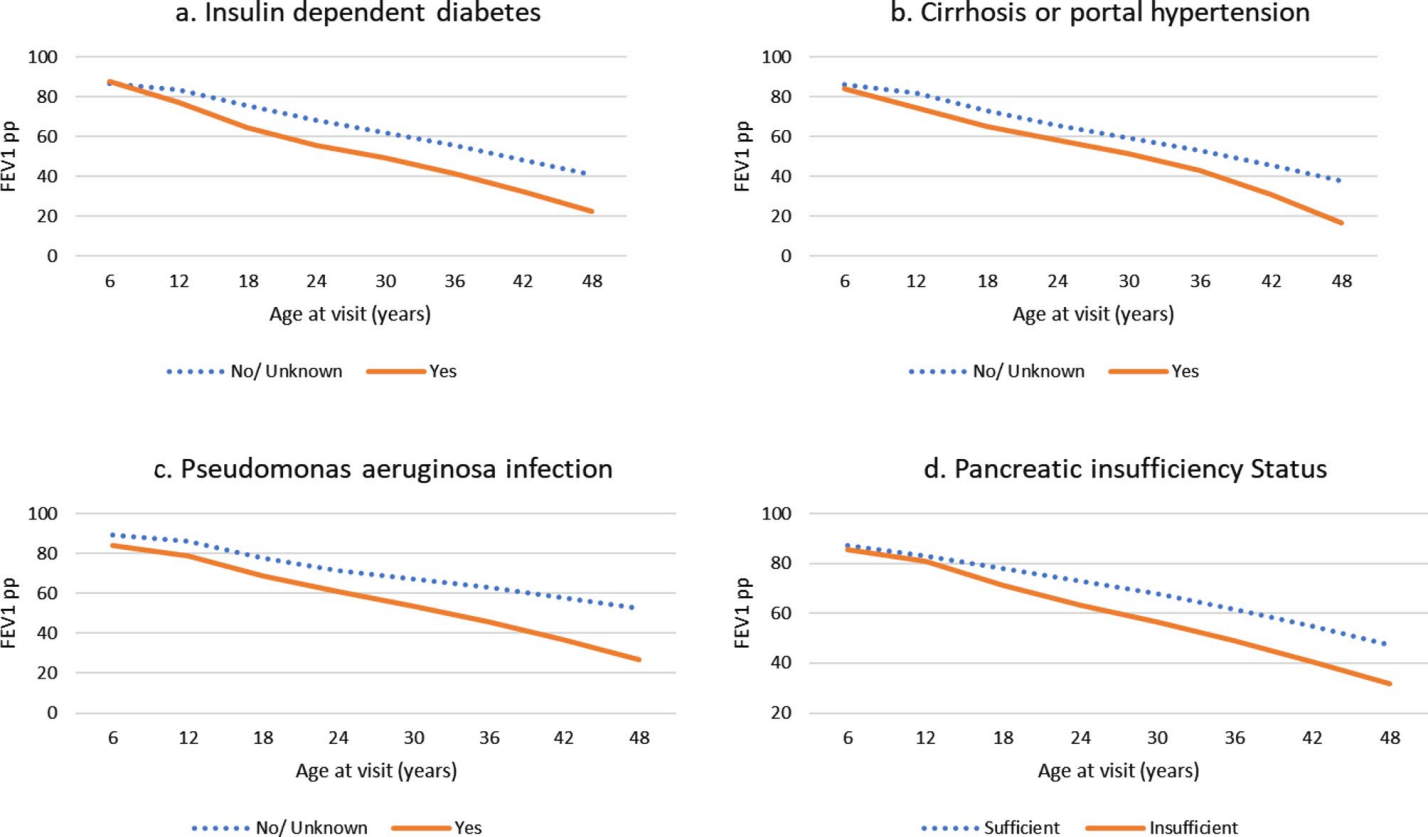
Decline in FEV_1_ percent predicted by key subgroups based on the final multivariate model.

In the multivariate analysis (Table 3), the following predictors were found to be statistically and independently associated with FEV_1_pp: age of visit, BMI z-score, lung transplantation and interaction between pre/post transplantation and age of visit, pancreatic insufficiency, insulin dependent diabetes, cirrhosis or portal hypertension, *Pseudomonas aeruginosa* infection and baseline variability in FEV_1_pp. The magnitude of the effect sizes was reduced as compared to the univariate results, probably due to confounding. Patients in the second, third and fourth quartile of BMI z-score had higher FEV_1_pp values of 3, 5, and 7 when compared to those in the first quartile (*p* < 0.001). Pancreatic insufficiency was associated with a lower FEV_1_pp value of − 3.7 (95% CI: − 5.5 to − 1.9) when compared to those who were sufficient (*p* < 0.001). Figure 2a–d highlights the predicted slope of FEV_1_pp across each level of covariate from the multivariate model. We observed that the slope of FEV1pp decline was steeper for patients with insulin dependent diabetes, cirrhosis or portal hypertension, *P. aeruginosa* infection and pancreatic insufficiency.

**Table 3:** Multivariate factors associated with FEV_1_ percent predicted.

Covariates	Coefficient	95% CI		*p* value
Restricted cubic spline, age of visit (6–12 years)	− 0.52	− 0.69	− 0.36	< 0.001
Restricted cubic spline, age of visit (13–18 years)	− 31.11	− 36.85	− 25.37	< 0.001
Restricted cubic spline, age of visit (19–30 years)	76.17	62.16	90.19	< 0.001
Restricted cubic spline, age of visit (> 30 years)	− 53.84	− 64.32	− 43.35	< 0.001
BMI z-score quartiles				
1st quartile	Reference			
2nd quartile	3.03	2.79	3.27	< 0.001
3rd quartile	5.28	4.99	5.57	< 0.001
4th quartile	6.89	6.53	7.24	< 0.001
Unknown	1.75	1.34	2.16	< 0.001
Lung transplant				
Yes (n = 351)	Reference			
No (n = 3300)	13.21	10.61	15.80	< 0.001
*Lung transplant and age of visit interactions*				
Restricted cubic spline, age of visit (6–12 years)*lung transplant (pre/post)	3.54	− 2.38	9.47	0.241
Restricted cubic spline, age of visit (13–18 years)*lung transplant (pre/post)	− 204.52	− 383.46	− 25.58	0.025
Restricted cubic spline, age of visit (19–30 years)*lung transplant (pre/post)	550.71	167.76	933.66	0.005
Restricted cubic spline, age of visit (> 30 years)*lung transplant (pre/post)	− 432.30	− 652.62	− 211.98	< 0.001
Insulin dependent diabetes				
No/unknown (n = 2988)	Reference			
Yes (n = 663)	− 5.70	− 7.52	− 3.87	< 0.001
Cirrhosis or portal hypertension				
No/unknown (n = 3415)	Reference			
Yes (n = 236)	− 3.42	− 6.10	− 0.74	0.012
Pancreatic insufficiency				
No (n = 710)	Reference			
Yes (n = 2726)	− 3.68	− 5.46	− 1.89	< 0.001
Unknown (n = 215)	− 2.61	− 6.26	1.04	0.161
*Pseudomonas aeruginosa* infection				
No/unknown (n = 1812)	Reference			
Yes (n = 1839)	− 4.73	− 6.17	− 3.29	< 0.001
Baseline variability in FEV_1_pp (n = 3651)	− 0.45	− 0.56	− 0.35	< 0.001

Joint modelling with mortality as the outcome showed that the results from the multivariate model generally persisted in terms of magnitude of effect as well as statistical significance (Supplementary Information Table S2). The effect of age of visit was less attenuated, while magnitude of effect for lung transplantation became larger when mortality as included in the model. There was a reduction in effect size for effects of comorbidities like diabetes and cirrhosis. The effect of pseudomonas and baseline variability in FEV_1_pp were hardly unchanged from the original analysis.

For the final multivariate model, the residuals were generally normally distributed, except for very slight skewness towards the tail ends of the distribution. As a sensitivity analysis, the model was re-run with the outliers (1% of observations of both ends of the distributions) excluded, and we found minimal changes to the magnitude of the coefficients (Supplementary Information Table S3). We found there was a less than 20% relative change in the magnitude of the coefficients for all variables except for cirrhosis or portal hypertension where there was a reduction in magnitude of the effect size (from − 3.4 to − 2.6). When lung transplantation covariate was excluded from the final multivariate model, minimal changes to the magnitude and direction of the effect sizes for most of the covariates in the final multivariate model were seen, except for pancreatic insufficiency (coefficient of − 4.6 versus − 3.7 in the original analysis) and baseline variability in FEV1pp (coefficient of − 0.55 versus − 0.45 in the original analysis).

## Discussion

Our study has identified a number of significant demographic and clinical predictor variables associated with FEV_1_pp among people with CF. We have addressed some existing methodological challenges particularly relating to accounting for changes in slope of FEV_1_pp post lung transplantation, dealing with non-linearity through restricted cubic splines and joint modelling with mortality as an outcome.

We found that gender was not significant in the multivariate model. In a systematic review, the effect of gender was mixed, with 5 out of the 9 articles finding a steeper decline in FEV_1_pp among females, particularly from age 14 onwards^4^. However, the review did not look at gender effects post 21 years of age, presumably due to lack of follow-up.

The effect of patient genotype on FEV_1_pp was also not consistent in literature with only 3 out of 8 studies looking at this factor demonstrating that the homozygous F508del mutation group had a greater decline in FEV_1_pp compared to the heterozygous A455E group, one study showing the effect in the opposite direction and others with no effect^4^. In our study, patients with pancreatic insufficiency had lower FEV_1_pp as compared to those who were sufficient, which was consistent with what has been reported in literature^4^. In our study, although patient genotype was significant in the univariate model, it was no longer significant in the multivariate model. The strong relationship between BMI and FEV1pp demonstrated in our results may not be due to obstruction, but rather poor growth/ nutrition.

We used FEV_1_pp as an endpoint for our study as calculated from the Global Lung Function (GLI) initiative^35^, although alternate older methods exist^36,37^. We used cubic splines to visually demonstrate the non-linear relationship between FEV_1_pp and age as well as to identify an optimal inflexion point in age where there is a significant change in slope for FEV_1_pp. Restricted cubic splines can greatly increase the power of these methods to model non-linear relationships. Conditional median survival age estimates from a recent study has shown increases of 11 and 13 years for males and females given survival to age 40 in F508del homozygotes, when compared to survival from birth^38^. This increase in conditional survival (survivorship bias) could explain the gradual decline in FEV_1_pp we found among our cohort of patients aged 40 years and above.

We also chose the linear mixed model over others such as the GEE and GLM as our main aim was to study individual patient trajectories in FEV_1_pp, while the other models are based on population-based averages. We coded our model to allow for separate slopes for patients before and after lung transplantation and we also extended the model to jointly analyse mortality as an outcome to account for any effect of censoring in the data.

Our registry does not collect data on physical findings and symptoms which have been shown to be risk factors for FEV_1_pp^11^ and hence these could not be included in the models. As an observational study, unmeasured predictors could affect our results, but our registry is designed according to best practice guidelines and recent research and fields are consistent with international registries. Our study also does not provide a formal comparison of the various statistical techniques used to study longitudinal changes in FEV_1_pp among patients with CF. Such an analysis would require simulation studies to establish the generalisability of results across different countries and settings. We have opted to include a category for patients with missing data for a particular variable and included them in the analysis so as to preserve the original sample size. Although techniques to impute missing data exist, they rely on the assumption that data is missing at random, which would be problematic to assume with our registry data, since one of the main aims of the registry is to provide benchmarked outcomes to sites and we rely on sites to provide us this data.

The major strength of our paper is that this study was based on a large multi-centre registry of 23 centres across the five states in Australia. By including patients who had lung transplant, we were also able to adequately address the issue of differences in FEV_1_pp slopes these patients could have before/ after transplantation as compared to other studies which opt to censor the data after lung transplantation^39^. We were also able to adequately account for the effect of mortality as an outcome rather than a factor variable through joint modelling. Our model also adequately allowed for Centre treatment effects as well as allowed individual patients to have their own starting point FEV_1_pp and trend over age at visit. We also undertook a sensitivity analysis to test the robustness of key assumptions, specifically the impact of outliers and excluding lung transplantation from the final model. Our population based study provides a unique opportunity to forecast individual patient-level trajectory of FEV_1_pp.

## Conclusion

Our study has addressed important methodological challenges in the analysis of FEV_1_pp among patients with CF and provide a holistic approach to analysis of such longitudinal data. This epidemiological modelling can better aid epidemiologists through providing a framework to understand the impact of new and potentially useful treatments and interventions.

## Supplementary information

Supplementary Information
