# Supplementary material for: Lung function over the life course of paediatric and adult patients with cystic fibrosis from a large multi-centre registry
Source: Sci Rep. 2020 Oct 15;10:17421. doi: 10.1038/s41598-020-74502-1 (PMC7567842; doi:10.1038/s41598-020-74502-1)
Supplement: Supplementary file 1 — Supplementary Information [file 41598_2020_74502_MOESM1_ESM.docx]

**Lung function over the life course of paediatric and adult patients with cystic fibrosis from a large multi-centre registry**

*Arul Earnest PhD^1^, Farhad Salimi PhD^1^, Claire E. Wainwright MBBS, MD, ^2,3^, Scott C. Bell MBBS, MD, ^2,4,5^, Rasa Ruseckaite PhD^1^, Tom Ranger PhD^1^, Tom Kotsimbos MBBS, MD, ^6^, Susannah Ahern MBBS, FRACMA, PhD^1^.

**Affiliations:**

^1^ School of Public Health and Preventive Medicine, Monash University, Melbourne

^2^ Faculty of Medicine, University of Queensland

^3^ Department of Respiratory and Sleep Medicine, Queensland Children’s Hospital, Brisbane

^4^ Translational Research Institute, Brisbane

^5^ Department of Thoracic Medicine, The Prince Charles Hospital, Brisbane

^6^ Department Respiratory Medicine, Alfred Hospital, Melbourne

*** Corresponding author:**

Associate Professor Arul Earnest

School of Public Health and Preventive Medicine, Monash University

Room 415, Level 4,

553 St Kilda Road,

Melbourne VIC 3004

Phone: +61 3 9903 0112/ Fax: +61 3 9903 0556

Email: [arul.earnest@monash.edu](mailto:arul.earnest@monash.edu)

**Appendix**

| **Table S1. Comparing models to deal with non-linearity between age of visit and FEV_1_pp.** | | |
| --- | --- | --- |
| **Number** | **Model** | **AIC** |
| 1 | Age of visit as linear | 723625 |
| 2 | Age of visit as categorical (6-12, 13-17, 18+) | 724084 |
| 3 | Age of visit as quadratic term | 723618 |
| 4 | Restricted cubic splines with 3 knots (9, 17, 37) | 723619 |
| 5 | Restricted cubic splines with 4 knots (8, 15, 22, 44) | 723479 |
| 6 | Restricted cubic splines with 5 knots (8, 13, 17, 26, 44) | 723479 |
| 8 | **Restricted cubic splines with knots defined at clinically meaningful locations (12, 18, 30)** | **723447** |

*AIC: Akaike Information Criterion*

| **Table S2. Multivariate factors associated with FEV_1_ percent predicted (joint modelling with survival outcome)** | | | | |
| --- | --- | --- | --- | --- |
| **Covariates** | **Coefficient** | **95 % CI** | | **p-value** |
| Restricted cubic spline, age of visit (6-12 years) | -1.19 | -1.29 | -1.09 | <0.001 |
| Restricted cubic spline, age of visit (13-18 years) | -8.81 | -12.75 | -4.87 | <0.001 |
| Restricted cubic spline, age of visit (19-30 years) | 25.34 | 15.92 | 34.75 | <0.001 |
| Restricted cubic spline, age of visit (>30 years) | -18.82 | -25.52 | -12.13 | <0.001 |
|  |  |  |  |  |
| BMI z-score quartiles |  |  |  |  |
| 1st Quartile | Reference |  |  |  |
| 2nd Quartile | 3.00 | 2.75 | 3.26 | <0.001 |
| 3th Quartile | 5.06 | 4.76 | 5.35 | <0.001 |
| 4th Quartile | 6.22 | 5.87 | 6.57 | <0.001 |
| Missing | 2.20 | 1.81 | 2.60 | <0.001 |
|  |  |  |  |  |
| Lung Transplant |  |  |  |  |
| Yes | Reference |  |  |  |
| No | 25.37 | 23.45 | 27.30 | <0.001 |
|  |  |  |  |  |
| *Lung transplant and age of visit interactions* |  |  |  |  |
| Restricted cubic spline, age of visit (6-12 years)*lung transplant (pre/post) | 4.54 | -1.11 | 10.19 | 0.115 |
| Restricted cubic spline, age of visit (13-18 years)*lung transplant (pre/post) | -211.49 | -394.12 | -28.86 | 0.023 |
| Restricted cubic spline, age of visit (19-30 years)*lung transplant (pre/post) | 538.86 | 142.77 | 934.96 | 0.008 |
| Restricted cubic spline, age of visit (>30 years)*lung transplant (pre/post) | -396.15 | -627.07 | -165.24 | 0.001 |
|  |  |  |  |  |
| Insulin dependent diabetes |  |  |  |  |
| No/unknown | Reference |  |  |  |
| Yes | -4.46 | -5.97 | -2.94 | <0.001 |
|  |  |  |  |  |
| Cirrhosis or portal hypertension |  |  |  |  |
| No/unknown | Reference |  |  |  |
| Yes | -2.17 | -4.48 | 0.15 | 0.067 |
|  |  |  |  |  |
| Pancreatic Insufficiency |  |  |  |  |
| No | Reference |  |  |  |
| Yes | -4.79 | -6.22 | -3.36 | <0.001 |
| Unknown | -0.68 | -3.29 | 1.92 | 0.608 |
|  |  |  |  |  |
| Pseudomonas aeruginosa infection |  |  |  |  |
| No/unknown | Reference |  |  |  |
| Yes | -4.71 | -5.87 | -3.55 | <0.001 |
|  |  |  |  |  |
| Baseline variability in FEV_1_pp | -0.46 | -0.54 | -0.37 | <0.001 |

| **Table S3. Multivariate factors associated with FEV_1_ percent predicted (excluding outliers)** | | | | |
| --- | --- | --- | --- | --- |
| **Covariates** | **Coefficient** | **95 % CI** | | **p-value** |
| Restricted cubic spline, age of visit (6-12 years) | -0.64 | -0.78 | -0.50 | <0.001 |
| Restricted cubic spline, age of visit (13-18 years) | -29.42 | -34.21 | -24.63 | <0.001 |
| Restricted cubic spline, age of visit (19-30 years) | 72.87 | 61.17 | 84.58 | <0.001 |
| Restricted cubic spline, age of visit (>30 years) | -52.38 | -61.21 | -43.55 | <0.001 |
|  |  |  |  |  |
| BMI z-score quartiles |  |  |  |  |
| 1st Quartile | Reference |  |  |  |
| 2nd Quartile | 2.61 | 2.42 | 2.80 | <0.001 |
| 3th Quartile | 4.46 | 4.23 | 4.69 | <0.001 |
| 4th Quartile | 6.09 | 5.81 | 6.38 | <0.001 |
| Missing | 1.86 | 1.53 | 2.19 | <0.001 |
|  |  |  |  |  |
| Lung Transplant |  |  |  |  |
| Yes | Reference |  |  |  |
| No | 13.38 | 10.74 | 16.01 | <0.001 |
|  |  |  |  |  |
| *Lung transplant and age of visit interactions* |  |  |  |  |
| Restricted cubic spline, age of visit (6-12 years)*lung transplant (pre/post) | 2.93 | -1.60 | 7.46 | 0.205 |
| Restricted cubic spline, age of visit (13-18 years)*lung transplant (pre/post) | -211.78 | -348.75 | -74.81 | 0.002 |
| Restricted cubic spline, age of visit (19-30 years)*lung transplant (pre/post) | 604.80 | 310.85 | 898.76 | <0.001 |
| Restricted cubic spline, age of visit (>30 years)*lung transplant (pre/post) | -505.94 | -676.51 | -335.36 | <0.001 |
|  |  |  |  |  |
| Insulin dependent diabetes |  |  |  |  |
| No/unknown | Reference |  |  |  |
| Yes | -5.92 | -7.81 | -4.03 | <0.001 |
|  |  |  |  |  |
| Cirrhosis or portal hypertension |  |  |  |  |
| No/unknown | Reference |  |  |  |
| Yes | -2.61 | -5.39 | 0.17 | 0.066 |
|  |  |  |  |  |
| Pancreatic Insufficiency |  |  |  |  |
| No | Reference |  |  |  |
| Yes | -4.01 | -5.86 | -2.17 | <0.001 |
| Unknown | -2.42 | -6.14 | 1.31 | 0.203 |
|  |  |  |  |  |
| Pseudomonas aeruginosa infection |  |  |  |  |
| No/unknown | Reference |  |  |  |
| Yes | -4.89 | -6.39 | -3.40 | <0.001 |
|  |  |  |  |  |
| Baseline variability in FEV_1_pp | -0.46 | -0.56 | -0.35 | <0.001 |

| **Table S4. Multivariate factors associated with FEV_1_ percent predicted (excluding lung transplantation)** | | | | |
| --- | --- | --- | --- | --- |
| **Covariates** | **Coefficient** | **95 % CI** | | **p-value** |
| Restricted cubic spline, age of visit (6-12 years) | -0.53 | -0.70 | -0.37 | <0.001 |
| Restricted cubic spline, age of visit (13-18 years) | -32.09 | -37.95 | -26.22 | <0.001 |
| Restricted cubic spline, age of visit (19-30 years) | 78.69 | 64.35 | 93.03 | <0.001 |
| Restricted cubic spline, age of visit (>30 years) | -53.43 | -64.21 | -42.66 | <0.001 |
|  |  |  |  |  |
| BMI Z-score quartiles |  |  |  |  |
| 1st Quartile | Reference |  |  |  |
| 2nd Quartile | 3.06 | 2.82 | 3.30 | <0.001 |
| 3th Quartile | 5.31 | 5.02 | 5.61 | <0.001 |
| 4th Quartile | 6.91 | 6.55 | 7.27 | <0.001 |
| Missing | 1.76 | 1.35 | 2.18 | <0.001 |
|  |  |  |  |  |
| Insulin dependent diabetes |  |  |  |  |
| No/unknown | Reference |  |  |  |
| Yes | -6.25 | -8.24 | -4.25 | <0.001 |
|  |  |  |  |  |
| Cirrhosis or portal hypertension |  |  |  |  |
| No/unknown | Reference |  |  |  |
| Yes | -3.45 | -6.40 | -0.50 | 0.022 |
|  |  |  |  |  |
| Pancreatic Insufficiency |  |  |  |  |
| No | Reference |  |  |  |
| Yes | -4.63 | -6.58 | -2.67 | <0.001 |
| Unknown | -2.21 | -6.15 | 1.73 | 0.272 |
|  |  |  |  |  |
| Pseudomonas aeruginosa infection |  |  |  |  |
| No/unknown | Reference |  |  |  |
| Yes | -5.00 | -6.56 | -3.44 | <0.001 |
|  |  |  |  |  |
| Baseline variability in FEV_1_pp | -0.55 | -0.66 | -0.43 | <0.001 |
